# Higher species richness enhances yield stability in intensively managed grasslands with experimental disturbance

**DOI:** 10.1038/s41598-018-33262-9

**Published:** 2018-10-09

**Authors:** Eamon Haughey, Matthias Suter, Daniel Hofer, Nyncke J. Hoekstra, Jennifer C. McElwain, Andreas Lüscher, John A. Finn

**Affiliations:** 1 Teagasc, Environment Research Centre, Johnstown Castle, Wexford, Ireland; 2 Agroscope, Forage Production and Grassland Systems, Reckenholzstrasse 191, CH-8046 Zürich, Switzerland; 3 School of Biology & Environmental Science, Earth Institute, O’ Brien Centre for Science, University College Dublin, Belfield, Dublin 4 Ireland; 4 ETH Zürich, Institute of Agricultural Sciences, Universitätstrasse 2, CH-8092 Zürich, Switzerland; 5 Present Address: Louis Bolk Institute, Kosterijland 3-5, 3981AJ Bunnik, The Netherlands; 6 Present Address: Botany Department, School of Natural Sciences, Trinity College Dublin, Dublin, Ireland

## Abstract

Climate models predict increased frequency and severity of drought events. At an Irish and Swiss site, experimental summer droughts were applied over two successive years to grassland plots sown with one, two or four grassland species with contrasting functional traits. Mean yield and plot-to-plot variance of yield were measured across harvests during drought and after a subsequent post-drought recovery period. At both sites, there was a positive relationship between species richness and yield. Under rainfed control conditions, mean yields of four-species communities were 32% (Wexford, Ireland) and 51% (Zürich, Switzerland) higher than in monocultures. This positive relationship was also evident under drought, despite significant average yield reductions (−27% at Wexford; −21% at Zürich). Four-species communities had lower plot-to-plot variance of yield compared to monoculture or two-species communities under both rainfed and drought conditions, which demonstrates higher yield stability in four-species communities. At the Swiss but not the Irish site, a high degree of species asynchrony could be identified as a mechanism underlying increased temporal stability in four-species communities. These results indicate the high potential of multi-species grasslands as an adaptation strategy against drought events and help achieve sustainable intensification under both unperturbed and perturbed environmental conditions.

## Introduction

Altered precipitation patterns and rising atmospheric temperatures are expected to cause an increase in the frequency and intensity of drought events^1^. Drought events have strong negative effects on the aboveground biomass (yield) of extensively and intensively managed grassland ecosystems^2–4^. Given the predicted increase in severe climate events and likely impacts on grasslands, grassland managers require practical adaptation strategies to maintain the quantity, quality and stability of forage yield. Under unperturbed conditions, multi-species swards have the potential to increase the sustainability of grassland-based agriculture without necessarily incurring any reductions in yield^5–7^. In extensively managed grasslands, higher yields are generally positively related to plant species richness (reviewed by Hooper *et al*.)^8^. Furthermore, the temporal stability of yield has also been positively related to species richness in extensively managed grasslands^9–12^; yet, under environmental perturbation, positive diversity effects on yield stability have only been partly evident^4,13,14^. In intensively managed grasslands, even modest increases species richness can result in strong yield benefits, when species are selected for complementary traits^5,15,16^; however, there are remarkably few examples relating diversity to yield stability, for unperturbed or perturbed conditions (but see^17,18^). This is despite the high economic importance of intensively managed grasslands and threats to food security that can be expected from climate change effects on grasslands.

In agricultural systems, the stable provision of yields is an important requirement for reliable farm-level income and also for global food security. A system with high yield stability has a mean yield that changes least in response to environmental variation over time and/or space^19^. In many studies of natural or semi-natural systems, analyses of temporal stability have used stability metrics such as the coefficient of variation^9^ (*σ*/*μ*) or its inverse^17,20^ (*μ*/*σ)*. While this approach is well established, even some of those who use it also acknowledge its limitations^21^. Carnus *et al*.^22^ clearly illustrated limitations of the use of CV for assessing stability of ecosystem function. As with any index value that is a composite of two or more variables, the values of a stability index (e.g. *μ/σ*) obscure the separate responses of mean and standard deviation, and a specific value can arise from multiple different values of *μ* and *σ*. In agricultural systems, a lower-yielding crop (low *μ*) can have high stability measured in this way, despite this crop not being a desirable option (where alternatives exist). For these reasons, we consider that both yield and yield standard deviation need to be jointly considered in an assessment of yield stability^11,22,23^. This joint approach is also useful for assessing the performance of different systems and adaptation strategies under perturbed environmental conditions.

**Figure 1:**
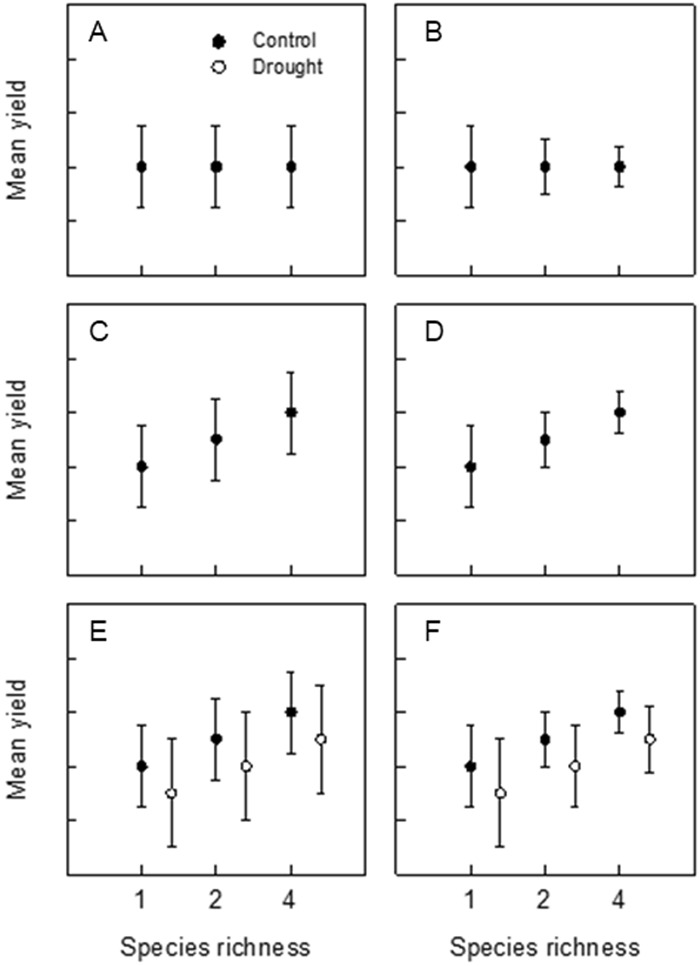
Hypothetical responses of yield and yield variance to increasing species richness under ambient conditions (**A**–**D**) and with an imposed disturbance (**E**,**F**). We expect that increasing species richness will result in higher yield due to facilitation and complementary interactions and lower variance due to compensation between species (**D**). In a system that undergoes a perturbation event, we expect reduced yield and increased variance (**E**). Ideally this decrease in yield and yield variance would be scaled in the same way as in scenario (**D**) to give the most desirable response (from an agricultural perspective) to drought (**F**).

Ideally, agricultural grasslands would produce high yields of good quality forage (high mean yield), and do so consistently (low yield variance) over variable growing conditions in time and space. A range of relationships between species richness, and mean and variance of yield are theoretically possible, which may result in enhanced, reduced or no effect on yield stability (Fig. 1; Wright *et al*.)^24^. For example, species richness may affect neither yield nor variance (Fig. 1A). More diverse communities may display decreased variance at equal yields (Fig. 1B). Interspecific interactions may increase mean yield (Fig. 1C) and yield stability would be considerably enhanced by increased yield and reduced yield variance (Fig. 1D). Under perturbed conditions, mean yield may be expected to decrease and yield variance increase (Fig. 1E). Positive relationships between richness and yield may persist under perturbed conditions (Fig. 1F), but this cannot be presumed.

In a related study, we recently demonstrated high drought resistance and/or resilience of mean yields of monocultures in an intensively managed grassland type^25^. Here, we investigated whether increased species richness (from one to four species) in intensively managed grassland enhanced mean yield and reduced yield variance over multiple harvests, and whether any such effects of species richness persist under an experimental perturbation that simulated a severe climate event (drought). Experimental summer droughts were applied to grassland plots at two sites (Wexford, Ireland and Zürich, Switzerland), in two successive years. Plots were sown with one, two or four agronomically important grassland species. Species were selected for their contrasting functional traits: a shallow-rooted grass (*Lolium perenne*), a deep-rooted forb (*Cichorium intybus*), a shallow-rooted legume (*Trifolium repens*) and a deep-rooted legume (*Trifolium pratense*). A strong emphasis was given to the assessment of yield variance in relation to the overall yield of monocultures and mixtures. Mean yield, plot-to-plot variance of yield, and the stability index S (*μ*/*σ*) were measured across harvests during drought and after a subsequent post-drought recovery period. Additionally, to investigate potential mechanisms underlying the observed levels of yield stability, an analysis of species asynchrony was conducted for the mixtures. We focus on species asynchrony because it is identified as one important driver of community stability^26^.

## Results

### Magnitude of drought stress differed due to pedo-climatic conditions

**Figure 2:**
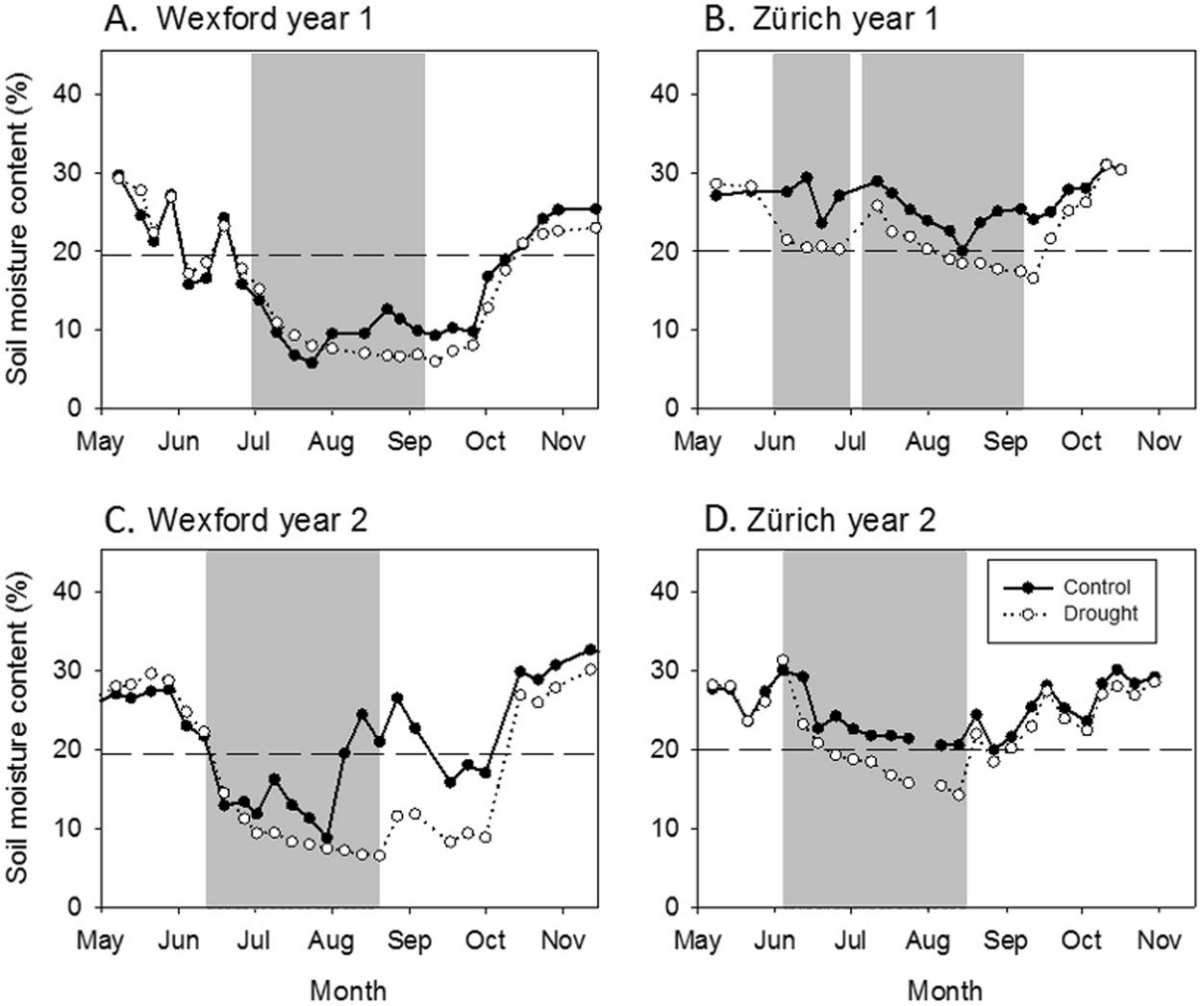
Weekly soil moisture content (%) at 10 cm depth at Wexford and 5 cm depth at Zürich (data are means, *n* = 3 replicates). Grey shading indicates the periods when rain was excluded from drought-treatment plots. The horizontal reference line is the soil moisture content that corresponds to a soil matric potential of −1.5 MPa, which is the approximate threshold of plant-available soil water. Note that the drought period had to be restarted at Zürich in year 1 after a heavy thunderstorm at the end of June.

Differences in treatment severities between sites were driven by soil physical properties as well as climatic conditions (Table 1 and Fig. 2). Relative to the 30-year mean, the summers of 2013 and, to a lesser extent, 2014 had lower precipitation than average for Wexford; −40.3% and −13.9%, respectively (Table 1 and Supplementary Fig. S1). Given the generally drier than average summers in Wexford, the experimental drought treatment resulted in extreme droughts in both years (Fig. 2). At Zürich, summer precipitation in the first experimental year considerably exceeded the 30-year average (+43.3%), which contributed to a less extreme drought than in Wexford. In year 2 at Zürich, the summer was drier than average (−33.3%), but the effect of the drought treatment was of a similar severity to the previous year (Fig. 2 and Supplementary Fig. S2). The effects of the rain-out shelters on plot-level microclimate was generally small (Supplementary Fig. S3).

**Table 1:** Climate data for both experimental sites including 30-year average climatic conditions.

Climatic metrics	Wexford			Zürich		
	30-year avg.	2013	2014	30-year avg.	2012	2013
Annual precipitation (mm)	1025	888	1138	1019	1165	1028
Summer precipitation (mm)^ǂ^	231	138	199	330	473	220
Summer precipitation (% of 30 year)^ǂ^	—	60	86	—	143	67
Total precipitation excluded (mm)	—	94	166	—	247	144
Annual precipitation excluded (%)	—	11	15	—	21	14
Summer precipitation excluded (%)	—	68	83	—	52	66
Mean annual temperature (°C)^†^	10.4	10.0	10.6	9.5	9.8	9.4
Mean summer temperature (°C)^†^	14.8	15.3	15.0	18.0	18.5	18.8

Notes: The 30-year average climate values were calculated for the period 1983 to 2012 at both sites from meteorological stations situated not more than 1.4 km from the experimental locations. ^†^Reference period for average temperature was from 1994 to 2012 at Wexford, ^ǂ^summer defined as June–August.

### Effects of drought on yield across harvests

**Figure 3:**
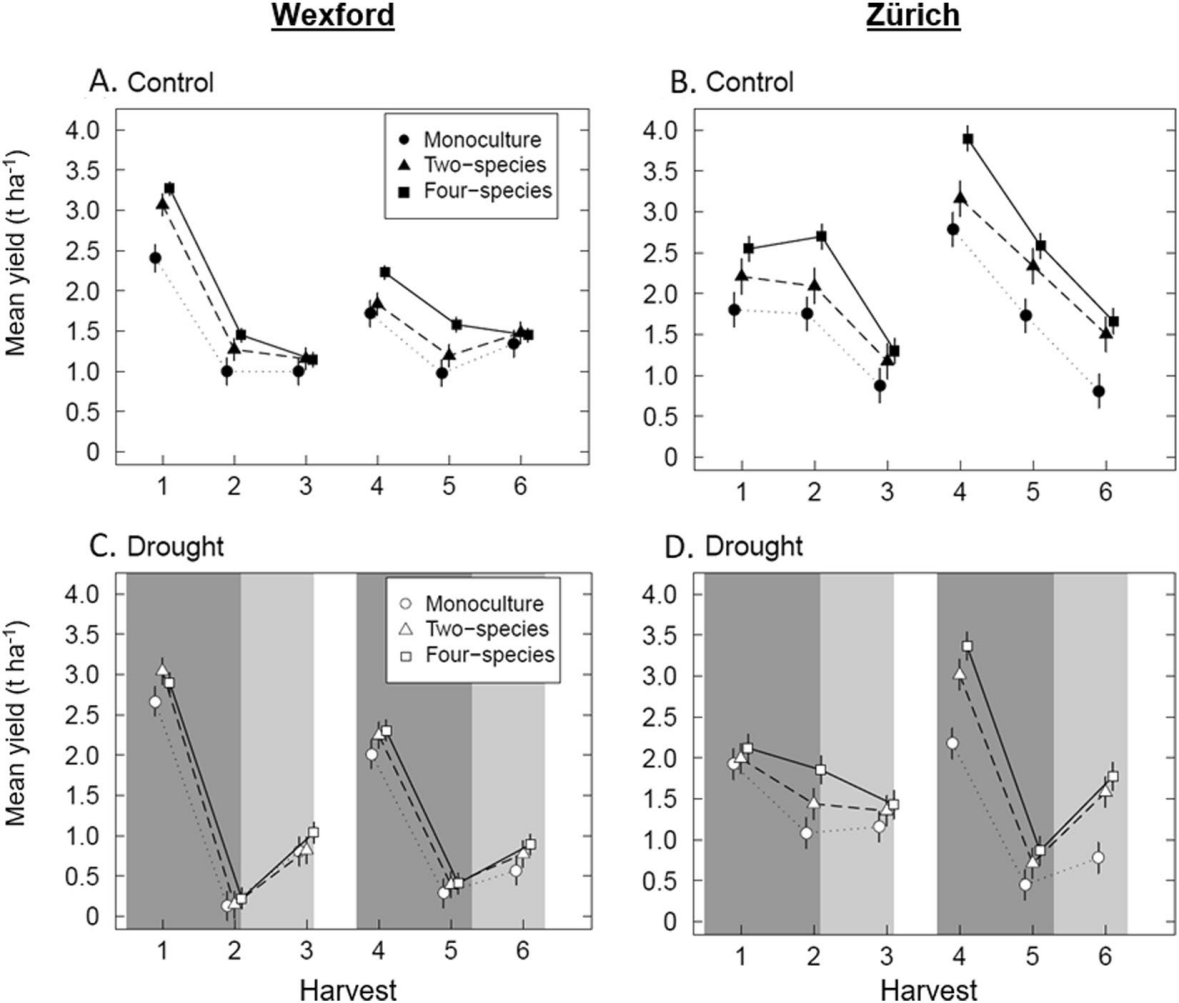
Effects of species richness and drought on yield for six harvests at the two sites. Mean yield and standard errors (aboveground biomass) of monocultures, two-species, and four-species mixtures under control conditions (**A**,**B**) and under drought (dark grey) and post-drought (light grey) periods (**C**,**D**) of two consecutive years (based on regression analysis, eqn. 1). At both sites, harvests 1–3 occurred in year 1 and harvests 4–6 occurred in year 2.

Across the six harvests, there were highly significant effects of species richness, drought and harvest on yields at both sites (Table 2 and Fig. 3). The greatest effect on yield by far was that of yield fluctuations across repeated harvests (main effect of harvest, Table 2, compare *F*-values). Changes in yield over harvests (on average across drought) were not modified by levels of species richness in Wexford, but were in Zürich (Fig. 3 and Table 2, richness × harvest interaction). However, based on the *F*-values, we conclude that the effect of species richness on yields of repeated harvests was relatively small (Wexford, *F* = 1.1, *P* < 0.326; Zürich, *F* = 4.7, *P* < 0.001).

**Table 2:** Summary of regression analysis of the effects of species richness (Richness), drought, and repeated harvests on aboveground biomass yield.

Variable	df_num_	df_den_	Wexford		Zürich	
			*F*-value	*P*	*F*-value	*P*
Richness level	2	32	23.3	<0.001	11.3	<0.001
Drought	1	32	122.0	<0.001	108.3	<0.001
Harvest	5	320	276.3	<0.001	182.4	<0.001
Richness × drought	2	32	2.3	0.118	1.7	0.190
Richness × harvest	10	320	1.1	0.326	4.7	<0.001
Drought × harvest	5	320	24.0	<0.001	34.7	<0.001
Richness × drought × harvest	10	320	1.0	0.435	0.7	0.710

Notes: df_num_, degrees of freedom of variable; df_den_, degrees of freedom of error.

Drought had the second largest effect on yield (Table 2). The extreme drought at Wexford in both years resulted in strong yield reductions across all diversity levels (Fig. 3). In contrast, at Zürich, the drought effect on yield was less severe in year one but substantial in year two (Fig. 3). Compared to the rainfed control, drought reduced the overall average yields (including the post-drought period) by −27% and −21% at Wexford and Zürich, respectively. With marginal richness × drought interactions at both sites (Table 2, *P* ≤ 0.190), the effect of drought over all harvests was reasonably consistent across the three levels of species richness.

### Species richness increased yield and reduced yield variance

**Figure 4:**
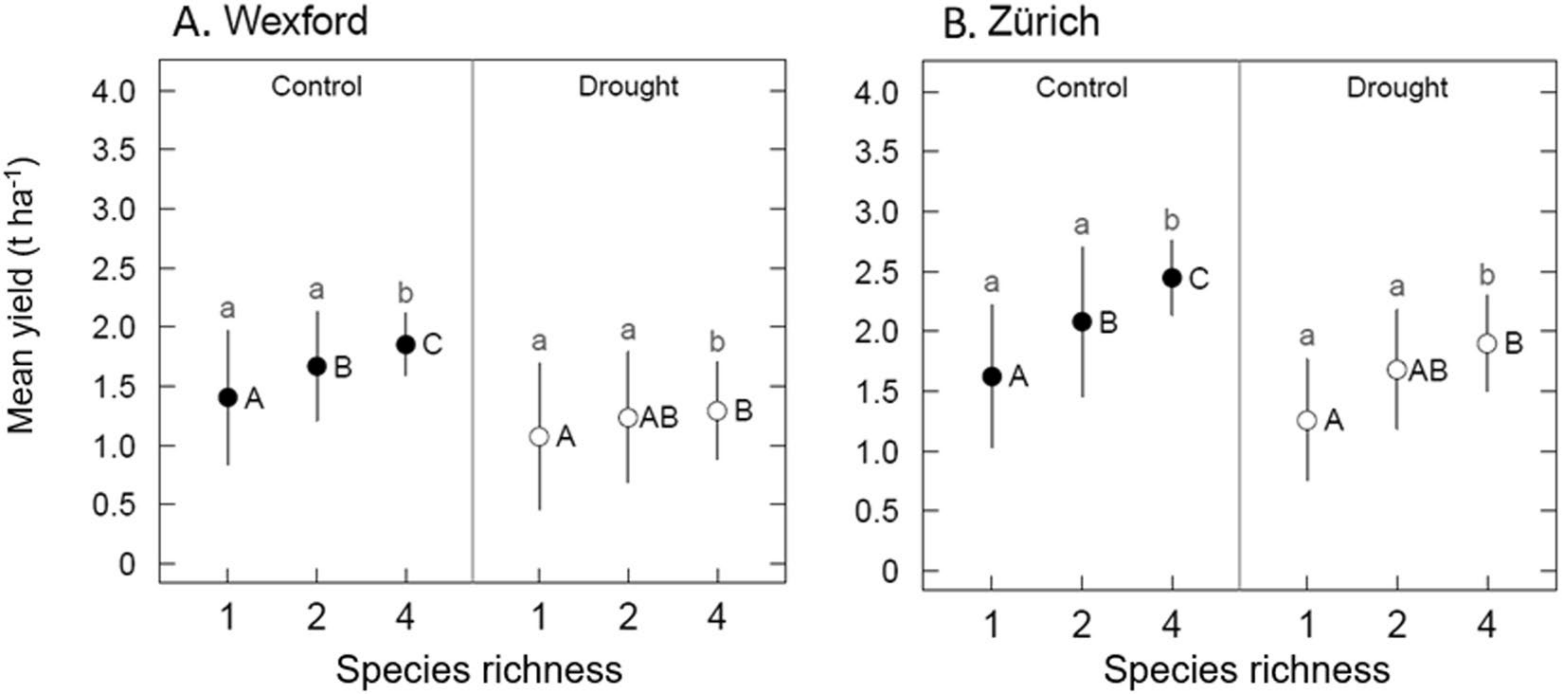
Effects of species richness and drought on yield mean and standard deviation across harvests under rainfed control and drought conditions at Wexford (**A**) and Zürich (**B**). Means are averaged across all six harvests. Standard deviations (SD) represent the plot-to-plot variation (see Methods) around the harvest means (*σ*, based on regression analysis, eqn. 1). Different letters indicate a difference at *P* < 0.05 based on regression analysis, except SD under drought at Zürich, which is at *P* < 0.1 (means: inference in black upper-case letters; SD: inference in grey lower-case letters).

Overall, under control and drought conditions at two different sites, species richness was positively related to yield (Table 2, *P* < 0.001 both sites) and negatively related to plot-to-plot standard deviation in yield (Fig. 4 and Supplementary Table S1). Under rainfed conditions, yields of four-species communities were 32% and 50% higher than the average of monoculture yields at Wexford and Zürich, respectively (Fig. 4). Under drought conditions, these diversity effects were 20% and 51% at Wexford and Zürich, respectively. The plot-to-plot standard deviation of yields was consistently and significantly lower in four-species mixtures compared to standard deviations in either the two-species mixtures or monocultures (Fig. 4).

**Figure 5:**
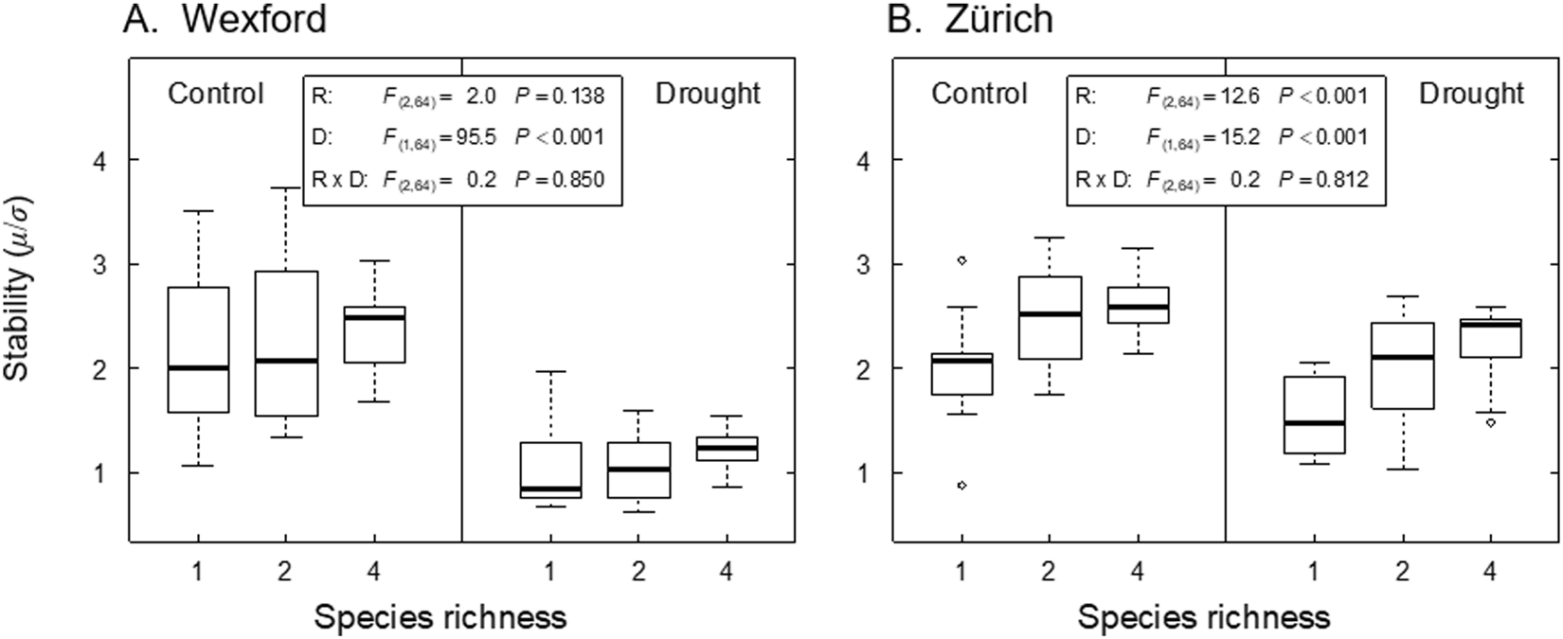
Effects of species richness (R) and drought (D) on yield stability at Wexford (**A**) and Zürich (**B**), under rainfed control and drought conditions. The stability index S = *μ*/*σ* was computed with *μ* and *σ* being the mean and the standard deviation across all harvests. Boxes are confined by the first and third quartile with the median in-between (bold). Whiskers extend to the most extreme data point that is no more than 1.5 times the interquartile range from the box.

In general, there was a positive species richness effect on values of the stability index S (*μ*/*σ*) under rainfed control and drought conditions at both sites (Fig. 5), with the effect being stronger at Zürich than at Wexford. Although there was a trend for increased values of S in four-species mixtures also at Wexford, relatively large yield variance in monocultures and two-species mixtures probably masked the positive richness effect on S (Fig. 5, compare interquartile ranges). Values of S within a site were reduced by drought at all richness levels (Fig. 5, significant drought effect and no richness × drought interactions).

**Figure 6:**
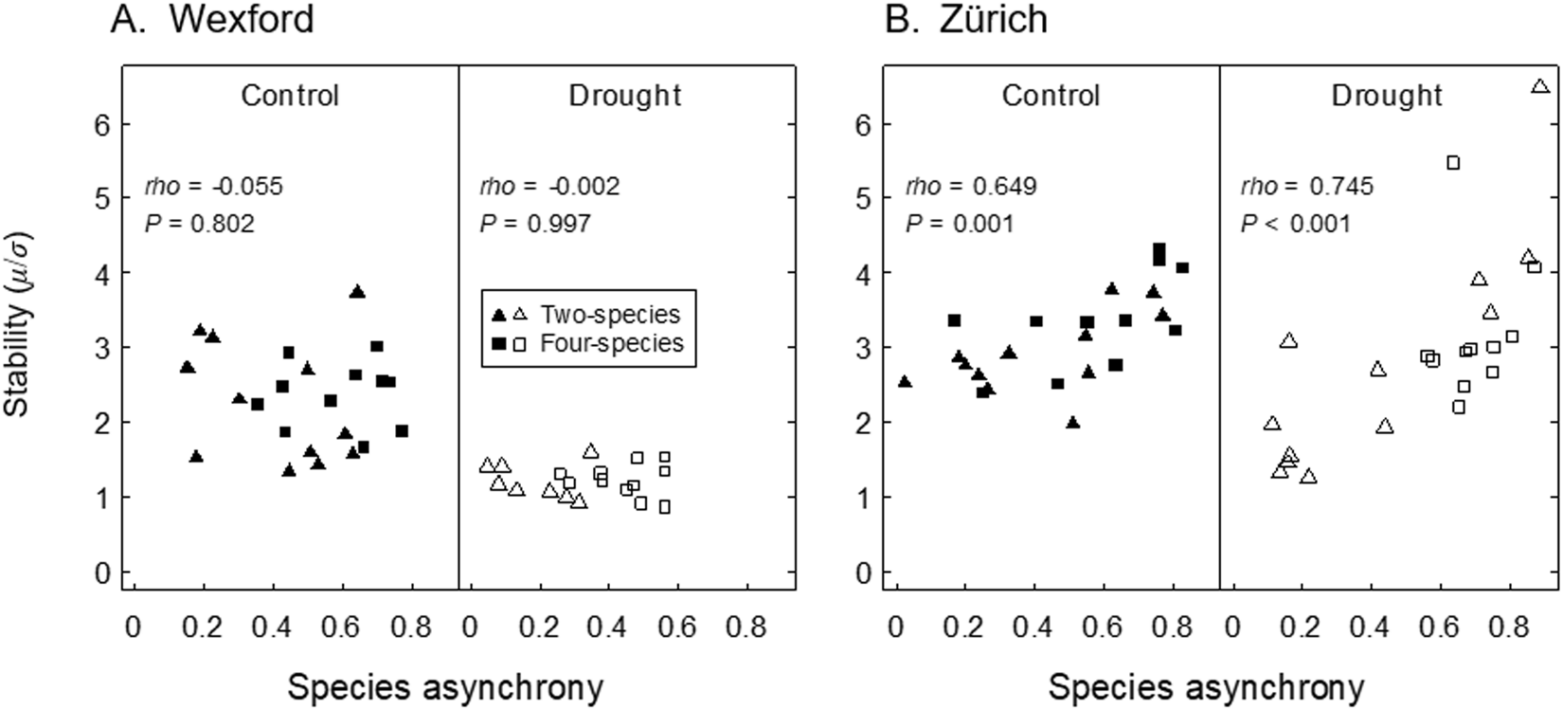
Relationship between species asynchrony and yield stability at Wexford (**A**) and Zürich (**B**) under rainfed control and drought conditions. The stability index S = *μ*/*σ* was computed with *μ* and *σ* being the mean and the standard deviation across all harvests. *rho*: spearman rank correlation. At Wexford under drought, four of the two-species mixtures had missing data for individual species (no asynchrony value computed).

A significantly positive correlation between the stability index S and species asynchrony was identified at Zürich under both control (*rho* = 0.649) and drought conditions (*rho* = 0.745, Fig. 6), but not in Wexford. At Wexford, however, species asynchrony itself was strongly reduced by drought (*P* < 0.001, Supplementary Table S2, Fig. 6), resulting in a distinctly reduced stability under drought at this site (Fig. 6, compare Fig. 5). Overall, while the asynchrony-stability correlation was driven by drought at Wexford (overall *rho* = 0.304, *P* = 0.051), the asynchrony-stability relationship was more affected by species richness at Zürich (richness effect on asynchrony: *P* < 0.001, Supplementary Table S2, Fig. 6).

## Discussion

At both sites, an increase in species richness from one to four species contributed to yield stability in intensively managed grassland communities. The mean yield of four-species communities over the sampled harvests was greater than the mean yields of monoculture and two-species communities. This is in agreement with other studies of grass-legume mixtures^5,15,16^. Importantly, plot-to-plot standard deviation of yield was also lower in the four-species communities. In our experiment, despite yield reductions due to drought, the positive richness effect observed in control conditions was still apparent under the drought treatments (Fig. 4), revealed by increased yields and reduced plot-to-plot standard deviation despite substantial seasonality in yields for all communities. Both sites experienced different severities of drought (severe and extreme), as well as different climatic and soil conditions (Table 1). Nevertheless, the relationship between species richness and both the mean and variance of yield were quite consistent. Overall, we demonstrate the role of higher species richness in promoting yield stability in intensively managed grassland communities in both control (rainfed) and perturbed (drought) conditions.

The assessment of yield stability is dominated in theory and practice by the testing of crop-by-environment interactions (e.g. Piepho^23^), and the stability of a crop or cropping system is assessed by measuring the variability of yields across varying environmental conditions. Therefore, in order to assess yield stability, it is vital that sufficient variation in environmental conditions is generated. Here, environmental variation was derived from two levels of water treatment, and inter-site differences in pedo-climatic conditions. Despite these differences, the observed relationships between species richness and forage yields were consistent. This indicates that the observed responses are relatively robust for these species and under an intensive management system, consistent with Craven *et al*.^13^.

The ability of species-rich communities to maintain ecosystem function under perturbed conditions can result from several mechanisms that include: (i) enhanced ecosystem performance due to synergistic interactions; (ii) the selection of species with improved performances, and; (iii) asynchronous responses among species to the perturbed environment^9,10,12,27,28^. Our choice of species was informed by an *a priori* selection of species with functional traits intended to maximise niche complementarity under the environmental conditions in the experiment. With agronomic performance clearly in mind, we intentionally chose agronomic cultivars of four species that were high-yielding, digestible by livestock (point ii, above), and had distinct functional traits expected to contribute to complementarity in resource utilisation^5,25^ (point i, above). Species with different rooting depths were expected to better utilise available soil resources (water and nutrients). Moreover, complementarity through the inclusion of legumes can increase yields through the supply of symbiotically fixed nitrogen^29^, and can act over a wide range of species proportions in mixtures^15,30^, which promotes stability of mixed communities. Synergistic interactions between legume and non-legume functional types have been shown for a variety of species and under very differing environmental conditions^5,30^, including drought^25^ and levels of N fertiliser^15,31^. In principle, the yield of multi-species communities is highly dependent on the selected composition of species, and this is most obvious in ecological experiments with random assembly of species e.g. Hector *et al*.^32^. In contrast, species’ selections of agronomic forage mixtures are decidedly non-random (see above), and different combinations of four-species agronomic mixtures comprising species with contrasting traits have consistently shown strong overyielding^5,15,25,30,33^. They might also reveal comparable results regarding the diversity-stability relationship, although this needs further investigation.

Regarding the improved performances of individual species under perturbed conditions (point ii, above), results were site-specific. Only monoculture communities of tap-rooting *C. intybus* displayed resistance to the extreme drought in year one at Wexford; in contrast, it was the other tap-rooted species *T. pratense* that displayed the most resistance at Zürich^25^. Despite the difference in drought responses between species at the two sites, there was a comparable effect of drought on yield of mixtures (Table 2 and Fig. 3). This in itself is indicative of the potential insurance effects of sowing such multi-species mixtures as an adaptation strategy against drought events.

Species asynchrony (point iii, above) can be an underlying mechanism that affects community temporal stability. Although species asynchrony was related to species richness at both sites, only at the Swiss site did this diversity effect translate to a clear asynchrony-stability correlation (Fig. 6B). In a similar system, Husse *et al*.^34^ also found that positive effects of species richness on the yield of intensively managed grassland were related to asynchronous seasonal growth patterns among species. However, at the Irish site in this study, species richness affected stability more through positive synergistic interactions between legume and non-legume species under both control and drought conditions^25,33,35^. Species’ synergistic interactions and asynchrony are not mutually exclusive and may act at the same time to enhance stability in more diverse communities. In addition, strong environmental perturbation such as the induced drought at the Irish site may affect stability more than does species richness or asynchrony (Figs 5A and 6A). Our results suggest that both species richness and environmental perturbation can have direct effects on stability or indirect effects *via* species asynchrony or other mechanisms^32^. Therefore, we infer that species asynchrony is one of several mechanisms that may contribute to the observed stability patterns here. Further research may clarify the role of asynchrony on stability under varying environmental conditions, which would also require measuring further environmental variables (e.g. soil nitrogen, light interception) in diversity-manipulation experiments.

The capacity to plan the species composition of intensively managed grasslands is one of several key differences between them and extensively managed grasslands^5^. Thus, because of such important differences, it should not simply be assumed that positive diversity effects observed in semi-natural and low-yielding grasslands will also be observed in intensively managed grasslands. Most experimental studies of grassland diversity and ecosystem function typically do not add nitrogen fertiliser, and observed responses between diversity and yield are within yield levels that would not be considered acceptable for more intensively managed grasslands. Yet, compared to less intensively managed systems, intensively managed grasslands can experience magnified yield losses and financial losses due to drought^4,36^. From both a scientific and economic perspective, it is therefore of high importance that we found positive diversity benefits on yield and yield variance in intensively managed grasslands, and did so under rainfed and drought conditions (Fig. 4).

The choice of stability metric is important. Here, results calculated using the S index (S = *μ*/*σ*; Fig. 5) displayed the same general trends as those in the analysis of means and associated standard deviations (Fig. 4). However, the effects of diversity did not appear to be as strong when analysed with S = *μ*/*σ*. This was to be expected, since the temporal yields of intensively managed grasslands, which are harvested multiple times per year, are known to be strongly affected by seasonality^37^, as was the case here (Table 2 and Fig. 3). In comparison, the measured effect of seasonality on semi-natural or natural grasslands that is based on one or two harvests per year would be expected to be absent or much lower, respectively. In such a case, a metric such as the coefficient of temporal variance would be subject to less variation and be more likely to detect differences among different communities in stability measured as *μ*/*σ*.

In agricultural systems that produce yields for food or forage, the choice of a community with a high value of S would not be sensible without due consideration of the mean yield of that community. For example, consider the mean and standard deviation of yields from crop A with *μ/σ* = 10/2.5 = 4 and crop B with *μ/σ* = 20/7 = 2.9. All else being equal, crop B has twice the yield of crop A and would be the preferred choice from these two, despite having lower stability as measured by *μ/σ*. In a case where a high value of S was accompanied by a low mean, the result would be a much lower level of food supply; in such a case, stability would be manifested by food supply being low, and reliably so. Looking at both the mean and variation of the response allows more informed decisions about such choices (see Carnus *et al*.)^22^. Here, we generally found greater mean values of yield as diversity increased, and lower (or not greater) levels of variation about the mean. Similar to the scenario depicted in Fig. 1F, this outcome is close to the best-case scenario for yield stability to be evident. In addition to the above caveats regarding the use of an S index, the stability index would be further confounded by the strong seasonality that drives high temporal variation in yield across all communities. When evaluating yield stability in intensively managed grasslands, this makes a strong case for examination of plot-to-plot variance in an analysis that accounts for high seasonal variation in yields.

Sustainable intensification confronts agricultural systems with the challenge of producing more while using fewer resources, protecting ecosystem services, and addressing the effects of climate change. Multi-species grasslands have the potential to improve the resource use efficiency of grassland forage production^5,15^. The increased forage yield of swards in which legumes are present is driven by niche complementarity and facilitative interactions occurring between legumes and other species^38^. Lüscher *et al*.^6^ listed the potential contribution of legumes to the key challenges of sustainable intensification as: (i) increasing forage yield, (ii) substituting inorganic N-fertiliser inputs with symbiotic N_2_ fixation, (iii) supporting mitigation and adaptation to climate change, and (iv) increasing the nutritive value and conversion efficiency of herbage. In addition to these arguments, our results show that higher plant diversity increased yield stability of forage production, even under drought events, and further highlight the potential of legume-based mixtures to contribute to sustainable intensification (see also Hofer *et al*.)^25^. Improved understanding of species-specific responses to severe weather events could help further improve species and cultivar selection for use in multi-species grasslands, and so better inform practical agricultural strategies to adapt grasslands to a climate with a higher frequency of severe events.

## Materials and Methods

A field experiment was established at two sites (i) Wexford, Ireland and (ii) Zürich, Switzerland (see Supplementary Appendix S1 and Hofer *et al*.^25^ for further information). The experiment was located on a soil of sandy-loam texture at Wexford and loam texture at Zürich. Four agricultural grassland species were selected based on a factorial combination of nitrogen-fixing (N-fixing) and root-depth traits; two non-fixing species, *Lolium perenne* L. (shallow-rooted grass) and *Cichorium intybus* L. (deep-rooted forb), and two N-fixing species, *Trifolium repens* L. (shallow-rooted legume) and *Trifolium pratense* L., (deep-rooted legume). Main-plots (5 m × 6 m) were sown following a simplex design (Supplementary Table S3), such that there were: monocultures of each of the four species, six binary combinations (50% of each of two species), an equi-proportional mixture (25% of each of the four species), and four-species mixtures dominated by each species in turn (79% of one species, 7% of the other three). There were three replicates of monocultures and the equi-proportional mixture, and two replicates of binary and dominated four-species mixtures (Supplementary Table S3). At each site, there was a total of 35 main-plots that were arranged according to a randomised incomplete block design.

### Drought treatment and site management

One year after establishment, a summer drought event of nine to ten weeks was simulated at each site over two years (2013 and 2014 at Wexford; and 2012 and 2013 at Zürich). During drought periods, precipitation was completely excluded from one randomly selected half (split-plot: 5 m × 3 m) of experimental main-plots using tunnel-shaped rain-out shelters (Supplementary Fig. S4). Microclimatic parameters were measured at a height of 80 cm, outside and underneath rain-out shelters during the drought treatment (Supplementary Fig. S3). At both sites, soil moisture content (SMC) was measured weekly in plots with equi-proportional mixtures, at two depths under control and drought conditions, and soil moisture desorption curves were determined from these plots to provide a common metric for the physical soil environment (see Supplementary Appendix S2). Aboveground biomass was harvested five times annually at Wexford and six times at Zürich, from a central strip of 5 m × 1.5 m in each split-plot. Harvests occurred approximately halfway through and at the end of each drought treatment, and again after six to eight weeks of post-drought recovery following the removal of rain-out shelters. Plots received mineral nitrogen (N) fertiliser at a rate of 130 kg N ha^−1^ year^−1^ (year 1) and 150 kg N ha^−1^ year^−1^ (year 2) at Wexford, and 200 kg N ha^−1^ year^-1^ in both years at Zürich. Both sites received phosphorus and potassium fertiliser applications in spring of each year following local fertiliser recommendations for intensively managed grassland. At each harvest, dry matter content (DMY) of each split-plot yield was determined by drying a subsample of the harvested fresh biomass. After two years, the subdominant species in two-species mixtures had at least 13% proportional contribution and all sown species were present in four-species mixtures, at both sites and under rainfed control and drought treatments (Supplementary Figs S5 and S6), meaning that none of the sown species died out after two years of experimental drought. More importantly, in four-species mixtures the potential for significant interactions between legume and non-legume species was sustained, as the summed proportions of each group were more than 14%.

### Data analysis

Analyses of the yield responses to experimental drought only included data from the mid-drought, end-of-drought and post-drought harvests in each year (the post-drought harvest comprised the first harvest after the removal of rain-out shelters). In intensively managed grassland systems with multiple harvests per year, large inherent yield differences among harvests are generally observed irrespective of levels of species richness^37^. Indeed, preliminary analyses revealed that yield differences among harvests over time masked the effects of richness on yield stability as assessed by an index such as S_temp_ = *μ*/*σ* (following Lehman and Tilman^20^, with *μ* and *σ* being the yield mean and standard deviation across all harvests). To better assess yield stability over time in this cropping system with multiple harvests per annum, we followed a two-stage approach. First, yield mean and variance among harvests were evaluated by linear mixed-effects regression^39^. With *y* being the yield response, the model was:

1 $${y}_{jkm}={\beta }_{1}\,{{\rm{Richness}}}_{j}+{\beta }_{2}\,{\rm{Drt}}\_{{\rm{Treat}}}_{k}+{\beta }_{3}\,{{\rm{Harvest}}}_{m}+{\lambda }_{1}\,{\rm{main}} \mbox{-} {\rm{plot}}+{\lambda }_{2}\,\mathrm{split} \mbox{-} \mathrm{plot}+{e}_{jk}$$

The fixed parameters *β* estimate aboveground biomass yield for species richness levels (factor ‘Richness’ with three levels *j*: monocultures, two-species mixtures and four-species mixtures), under rainfed control and drought conditions (factor ‘Drt_Treat’ with two levels *k*: control, drought), and over multiple harvests (factor ‘Harvest’ with six levels *m*: three harvests by two years). All two-way interactions and the three-way interaction among these fixed variables were included. The random variable ‘main-plot’ was included due to the multi-level structure of the design, i.e. to account for potential correlation of pairs of plots (one being a control, the other under drought), and the variable ‘split-plot’ (also random) was included to account for correlation of multiple harvests of each split-plot over time. Both *λ*_1_ and *λ*_2_ were modelled with *λ ~ N(0, σ*^2^). Using conditional *F*-tests^39^, eqn 1 directly evaluates the mean and variance of yield among harvests. Inference on differences among factor levels was derived from the model contrasts.

Second, to assess the plot-to-plot variance of yield across harvests as affected by species richness and the drought treatment, the residual variance parameter in eqn. 1 was defined as Var*(e*_*jk*_) = *σ*^2^*δ*_*jk*_^2^, with *δ* being a ratio to represent *j* × *k* variances, one for each of three species richness levels *j* under control and drought conditions *k* (i.e. six variances; see Pinheiro and Bates^39^ for details). Inference on differences among variances *e*_*jk*_ was quantified by likelihood ratio tests. Thus, this procedure allowed assessment of the plot-to-plot variance in relation to the overall yield once the very large temporal differences in (mean) yield among harvests were accounted for. Mean yields across harvests *m* for richness levels *j* at treatment conditions *k* were computed with ${\mu }_{jk}=1/m{\sum }_{1}^{m}{y}_{jkm}$, with *y*_*jkm*_ being the fixed estimates from eqn 1, while the corresponding standard deviation was ${\sigma }_{jk}=\sqrt{{\rm{Var}}({e}_{jk})\,}$. Restricted maximum likelihood was used for fixed parameter and variance estimation.

To compare our approach with a commonly used measure of stability in ecological studies, a stability index was calculated with S = *μ*/*σ*, with *μ* and *σ* being yield mean and standard deviation across all harvests on the plot level (see Lehman and Tilman)^20^. Analysis of variance (ANOVA) with factors ‘Richness’ and ‘Drt_Treat’, including their interaction, was used to derive inference on the index S (natural log transformed for analysis). Finally, we determined community-wide species asynchrony in mixtures^40^ to identify a potential mechanism underlying the observed diversity-stability relationship. Species asynchrony (1 − Φ) is defined by:

$$1-{\rm{\Phi }}=1-{\sigma }_{tot}^{2}\,/{({\sum }_{i=1}^{s}{\sigma }_{i})}^{2}$$

where Φ is species synchrony^40^, ${\sigma }_{tot}^{2}$ is the temporal variance of the community aboveground biomass, and *σ*_*i*_ is the temporal standard deviation in the aboveground biomass of species *i* in a community with *s* species. At Zürich, data for individual species biomass was missing for one harvest, meaning that Φ was computed from the remaining five harvests. The effect of ‘Richness’ and ‘Drt_Treat’ on species asynchrony was tested with ANOVA, while the spearman rank correlation *rho* was computed to evaluate the asynchrony-stability relationship. All data analysis was performed using the statistical software R^41^ (see Supplementary Appendix S3).

## Electronic supplementary material

Supplementary Information

## Data Availability

The yield data generated during and/or analysed during the current study are available in the Dryad Digital Repository at 10.5061/dryad.cq5h55f.
