# Supplementary material for: Higher species richness enhances yield stability in intensively managed grasslands with experimental disturbance
Source: Sci Rep. 2018 Oct 9;8:15047. doi: 10.1038/s41598-018-33262-9 (PMC6177466; doi:10.1038/s41598-018-33262-9)
Supplement: Supplementary file 1 — Supplementary Information [file 41598_2018_33262_MOESM1_ESM.pdf]

Supplementary Information

Higher species richness enhances yield stability in intensively managed grasslands with experimental disturbance

Eamon Haughey[1,3,6], Matthias Suter[2], Daniel Hofer[2,4], Nyncke J. Hoekstra[2,5], Jennifer C. McElwain[3,6], Andreas Lüscher[2] and John A. Finn[1*]

[1] Teagasc, Environment Research Centre, Johnstown Castle, Wexford, Ireland

[2] Agroscope, Forage Production and Grassland Systems, Reckenholzstrasse 191, CH-8046 Zürich, Switzerland

[3] School of Biology & Environmental Science, Earth Institute, O' Brien Centre for Science, University College Dublin, Belfield, Dublin 4, Ireland

[4] ETH Zürich, Institute of Agricultural Sciences, Universitätstrasse 2, CH-8092 Zürich, Switzerland

[5] Present address: Department of Experimental Plant Ecology, Radboud University Nijmegen, Heyendaalseweg 135, 6525 AJ Nijmegen, The Netherlands

[6] Present address: Botany Department, School of Natural Sciences, Trinity College Dublin, Dublin, Ireland.

Appendix S1 Extended site and experimental information

The experiment was established in two sites: (i) Johnstown Castle, Wexford, in the south-east of Ireland (52° 17' 35" N, 6° 30' 8" W, 58 m a.s.l.); and (ii) Reckenholz, Zürich, in the North of Switzerland (47° 26' 12" N, 8° 31' 51" E, 479 m a.s.l.). At Wexford, the experiment was located on a soil classified as brown earth of sandy-loam texture (top soil 32% silt, 12% clay, pH = 4.9) with an Atlantic-temperate climate, and at Zürich on a brown earth of loam texture (top soil 42% silt, 26% clay, pH = 7.0) with a continental climate (Table 1, main text). Four agricultural grassland species were selected based on the factorial combination of nitrogen-fixing (N-fixing) and root-depth traits; two non-fixing species, *Lolium perenne* L. (shallow-rooted grass, cultivar (cv.) Alligator) and *Cichorium intybus* L. (deep-rooted forb, cv. Puna II), and two N-fixing species, *Trifolium repens* L. (shallow-rooted legume, cv. Hebe) and *Trifolium pratense* L., (deep-rooted legume, cv. Dafila).

Drought treatment

A summer drought event of nine to ten weeks was simulated at each site over two years (2013 and 2014 at Wexford; 2012 and 2013 at Zürich). During drought periods, precipitation was excluded completely from one randomly selected split-plot in each experimental main-plot using tunnel-shaped rain-out shelters (see Fig. S4). Shelters consisted of steel frames (3 m × 5.5 m and a height of 140 cm) covered with a transparent plastic foil (SunMaster SuperThermic, 150 µm, XL Horticulture, UK, at Wexford and Gewächshausfolie UV5, 200 µm, Folitec Agrarfolien-Vertrieb, Germany, at Zürich). To ensure minimal changes to ambient temperature and relative humidity, shelters had a ventilation opening of 35 cm over the entire length at the top and at both

sides at the bottom and were left open at each end. At Zürich in year one, the drought period had to be restarted after 5 weeks due to a heavy thunderstorm.

Microclimatic parameters were measured under rainfed control and drought conditions during the drought period (Fig. S3). Relative humidity and air temperature were measured every 10 minutes at Wexford (temperature and relative humidity sensor, Voltcraft, Switzerland) and hourly at Zürich (temperature and relative humidity sensor, Decagon, US). Precipitation and other meteorological data were collected by respective national meteorological stations at a maximum distance of 1.4 km from each site.

Site management

Harvesting and fertiliser applications at each site were conducted in accordance with local management practices. Aboveground biomass was harvested five and six times annually at Wexford and Zürich, respectively, from a central strip of 5 m × 1.5 m in each split-plot. Biomass was cut at a height of 5 cm in Wexford using a Haldrup plot combine (HALDRUP GmbH., Germany) and at 7 cm in Zürich using a Hege 212 plot harvester (Wintersteiger GmbH, Austria). Dry matter content of each plot yield was determined by drying a subsample of bulk mass until constant weight (40 °C for 72 h for Wexford samples and at 100 °C for 24 h for Zürich samples).

Appendix S2 Soil moisture content measurements and determination of the threshold of plant-available soil water

At both sites soil moisture content (SMC) was measured weekly in the plots with equi-proportional mixtures at two depths under control and drought conditions. Measurements were recorded at 10 cm and 40 cm soil depth at Wexford (*n* = 3 per depth, PR2 Probe, Delta-T Devices, Cambridge, UK), and at 5 cm and 40 cm soil depth at Zürich (*n* = 3 per depth, 5TM sensor, Decagon, USA) under drought and control conditions (Figs. 2, S2). While useful, one limitation of SMC is that this metric does not reflect the physical characteristics of the soil[2,3]. To permit more informative intra- and inter-site comparisons of drought effects and severity, the approximate threshold of plant-available soil water was determined at both sites. Intact soil-cores (5 cm diameter) were removed from each of the three plots with equal species proportions under rainfed control and drought conditions at 10 and 40 cm deep (*n* = 2 replicates per depth). Using a standardised pressure plate method[4], soil water retention curves (the relationship between SMC and soil matric potential) were determined for each of these plots and soil depths, providing a metric to quantify water stress. For comparisons of drought effects, we refer to a soil matric potential of -1.5 MPa because this value gives an estimation of the lower limit to which a plant can extract water from a specific soil, and varies depending on soil physical and chemical characteristics. In our experiment, differences between sites at the threshold of -1.5 MPa were strongly driven by soil physical properties, particularly at 40 cm depth (Fig. S2), which the SMC alone does not reflect. This highlights the efficacy of including measurements of the soil matric potential to aid quantification of drought stress in accompaniment to SMC measurements[2]. Fertiliser was split into four and six applications per year in Wexford and Zürich, respectively.

References

1 Cornell, J. A. in *A Primer on Experiments with Mixtures* 23-93 (Wiley Online Library, 2002).

2 Vicca, S. *et al*. Urgent need for a common metric to make precipitation manipulation experiments comparable. *New Phytologist* **195**, 518-522 (2012).

3 Whalley, W. R., Ober, E. S. & Jenkins, M. Measurement of the matric potential of soil water in the rhizosphere. *Journal of Experimental Botany* **64**, 3951-3963 (2013).

4 Agroscope Reckenholz-Tänikon ART. Referenzmethoden der Forschungsanstalten, Band 2: Bodenuntersuchungen zur Standort-Charakterisierung. Agroscope Reckenholz-Tänikon ART, Zürich, Switzerland (2012).

Table S1 Summary of mean yields and standard deviations (SD, in parentheses) across all six harvests. Mean yields (μ_{jk}) and corresponding SDs (σ_{jk}) for monocultures, two-species mixtures, and four-species mixtures were calculated based on regression analysis (eqn. 1, with *j* and *k* as defined in the main text). Note that this data is a reorganisation of the data presented in Fig. 4. For richness levels within each of the site x treatment combinations, values with different superscripts (yield: upper case letters; SD: lower case letters) are significantly different at $P < 0.05$, except SD under drought at Zürich, which is at $P < 0.1$.

	**Mean yield (t ha^{-1})**			
	**Wexford**		**Zürich**	
**Group**	**Control**	**Drought**	**Control**	**Drought**
Monoculture	1.41^{A} (0.56^{a})	1.08^{A} (0.61^{a})	1.63^{A} (0.59^{a})	1.26^{A} (0.50^{a})
Two-species	1.67^{B} (0.46^{a})	1.24^{AB} (0.55^{a})	2.08^{B} (0.62^{a})	1.68^{B} (0.49^{a})
Four-species	1.86^{C} (0.26^{b})	1.30^{B} (0.41^{b})	2.45^{C} (0.31^{b})	1.90^{B} (0.40^{b})

Table S2 Summary of analysis of variance of the effects of species richness (Richness) and drought on species asynchrony.

		Wexford		Zürich	
**Variable**	**df**	***F*-value**	***P***	***F*-value**	***P***
Richness level	1	18.2	< 0.001	10.1	0.003
Drought	1	15.4	< 0.001	0.8	0.388
Richness x drought	1	0.7	0.410	0.8	0.384
Residuals	42				

Notes: Monocultures are omitted; df, degrees of freedom; at Wexford, df of residuals was 38 because of missing data for individual species in four plots under drought

Table S3 Species' proportions in experimental communities following a simplex design (see Cornell [1]). There were 15 plant communities that differed in the relative proportions of seed mass at sowing. Seed mass in monoculture was based on local practice. Communities differed in number of replicates (Rep) and species richness (SR). Mono: monocultures, Bi: binary mixtures of the two specified species, Equi-proportional: four-species mixtures sown with equal abundance of each species, Dom: four-species mixtures dominated by the specified species, Treat: drought treatment, Ctr: rainfed control, Drt: drought.

**Community**	**Sward**	***Lolium perenne* (Lp)**	***Cichorium intybus* (Ci)**	***Trifolium repens* (Tr)**	***Trifolium pratense* (Tp)**	**Treat**	**Rep**	**SR**
1	Mono Lp	1	0	0	0	Ctr, Drt	3	1
2	Mono Ci	0	1	0	0	Ctr, Drt	3	1
3	Mono Tr	0	0	1	0	Ctr, Drt	3	1
4	Mono Tp	0	0	0	1	Ctr, Drt	3	1
5	Equi-proportional	0.25	0.25	0.25	0.25	Ctr, Drt	3	4
6	Dom Lp	0.79	0.07	0.07	0.07	Ctr, Drt	2	4
7	Dom Ci	0.07	0.79	0.07	0.07	Ctr, Drt	2	4
8	Dom Tr	0.07	0.07	0.79	0.07	Ctr, Drt	2	4
9	Dom Tp	0.07	0.07	0.07	0.79	Ctr, Drt	2	4
10	Bi Lp-Ci	0.5	0.5	0	0	Ctr, Drt	2	2
11	Bi Lp-Tr	0.5	0	0.5	0	Ctr, Drt	2	2
12	Bi Lp-Tp	0.5	0	0	0.5	Ctr, Drt	2	2
13	Bi Ci-Tr	0	0.5	0.5	0	Ctr, Drt	2	2
14	Bi Ci-Tp	0	0.5	0	0.5	Ctr, Drt	2	2
15	Bi Tr-Tp	0	0	0.5	0.5	Ctr, Drt	2	2

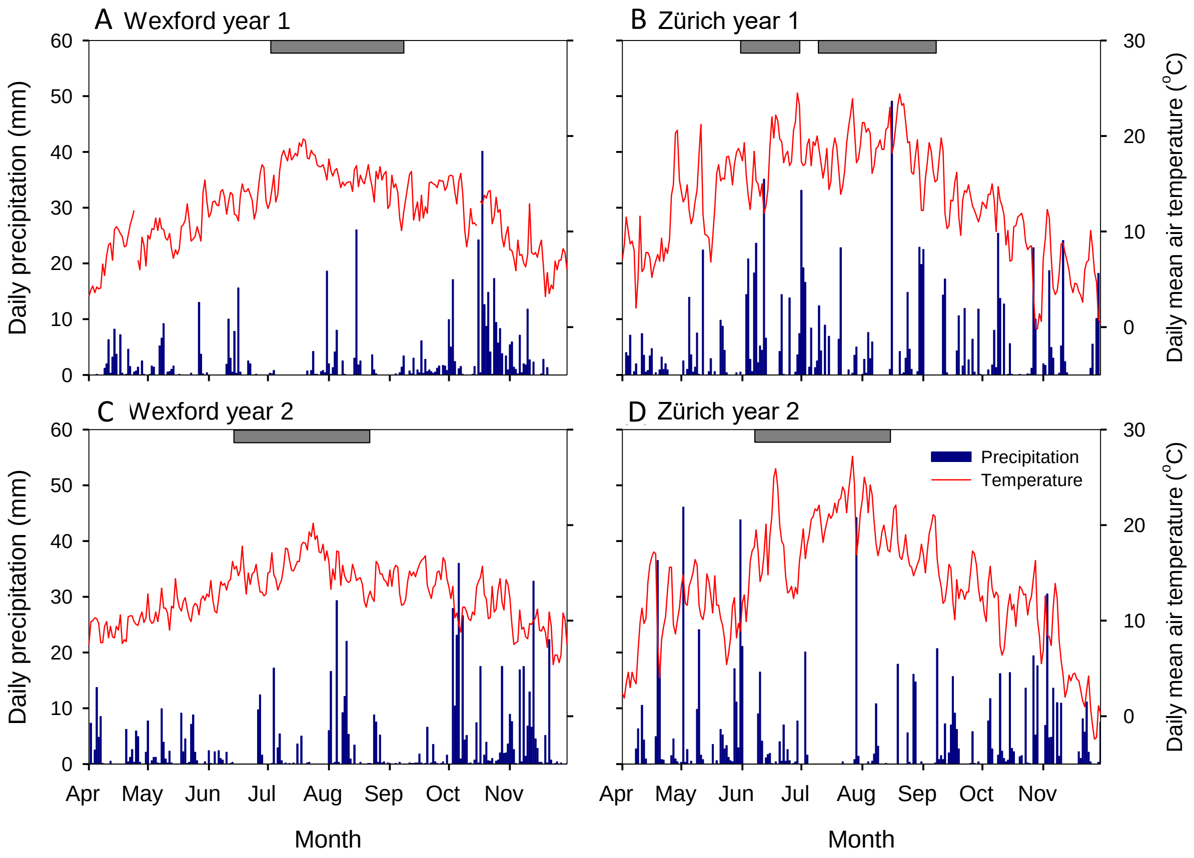

Fig. S1 Meteorological data from April to November in: 2013 and 2014 at Wexford (a, c); and 2012 and 2013 at Zürich (b, d). Data are from respective national meteorological stations located at a maximum distance of 1400 m from each site. Horizontal grey bars indicate the periods when rain was excluded from drought-treatment plots.

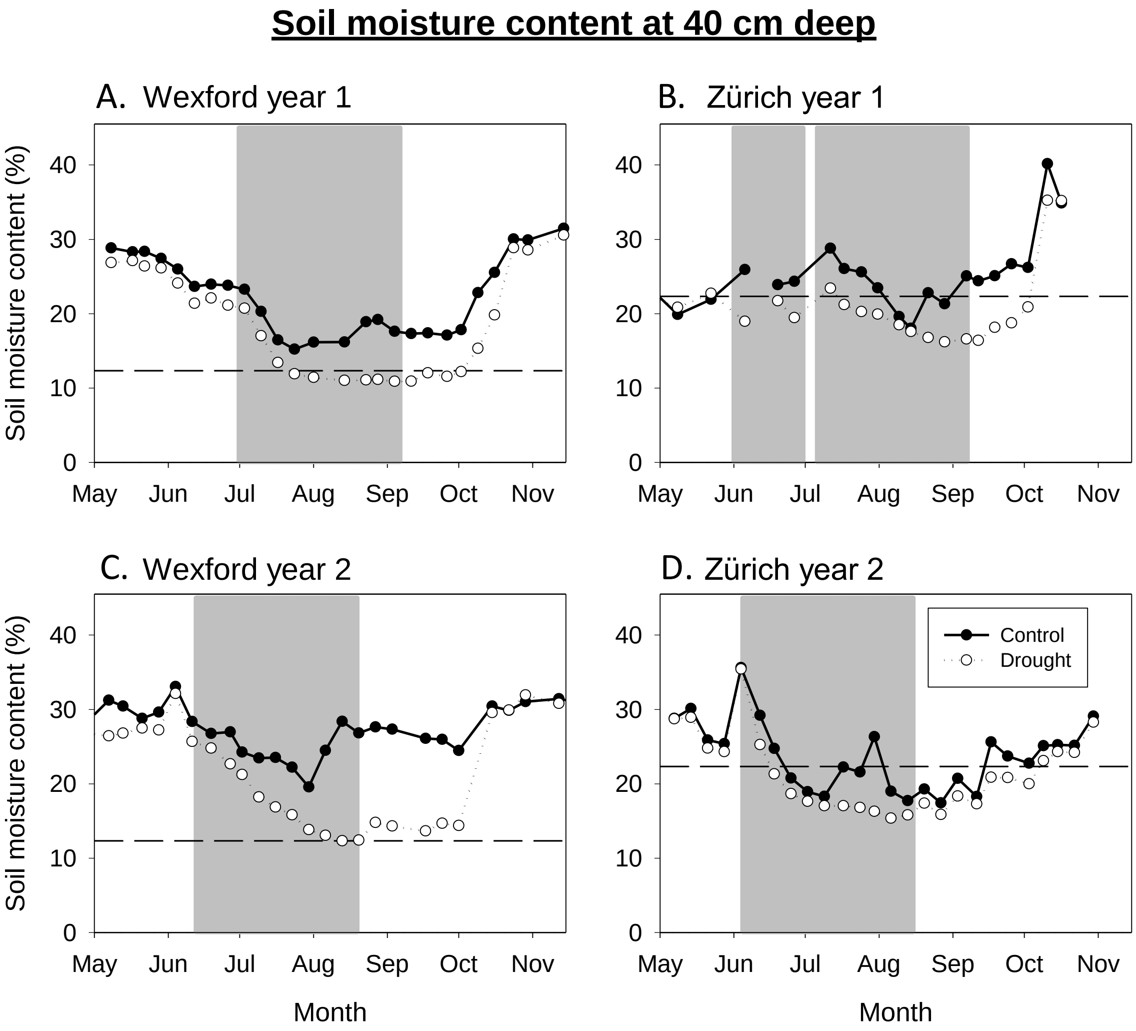

Fig. S2 Weekly soil moisture content (SMC) at 40 cm deep (data are means, n = 3 replicates). Grey shading indicates the periods when rain was excluded from drought-treatment plots. The horizontal reference line is the soil moisture content that corresponds to a soil matric potential of -1.5 MPa, which is the approximate threshold of plant-available soil water.

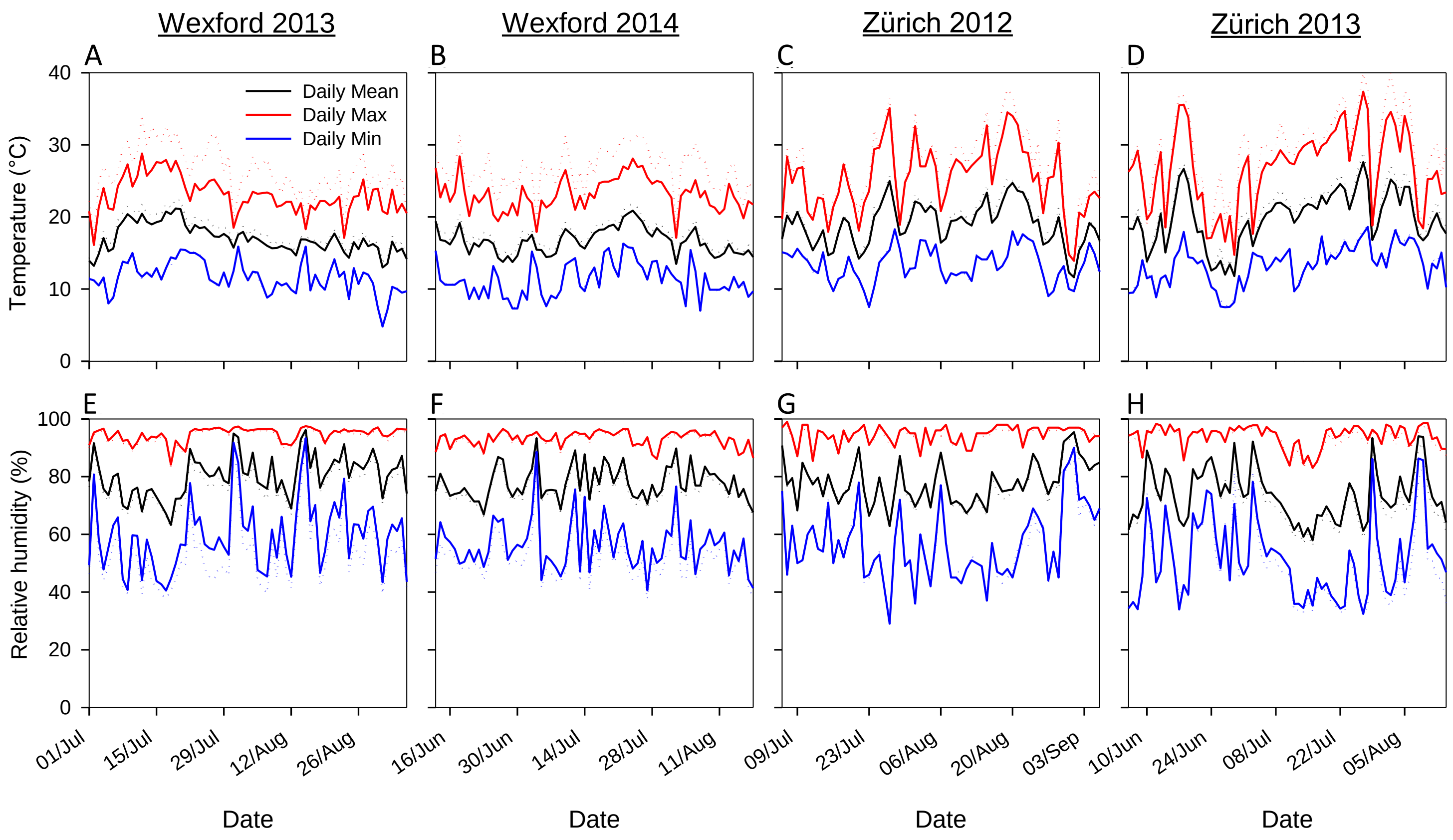

Fig. S3 Micro-climate data for underneath (dashed lines) and outside (solid lines) rain-out shelters, during the drought treatment period at Wexford and Zürich in both years of the experiment. In year 1 mean daily temperature was 0.83 °C and 0.46 °C higher and mean daily relative humidity was 2.23% and 0.69% lower under shelters compared to control plots at Wexford and at Zürich, respectively. Photosynthetic photon flux density above vegetation canopy was well beyond the threshold of light saturation of photosynthesis underneath and outside rain-out shelters.

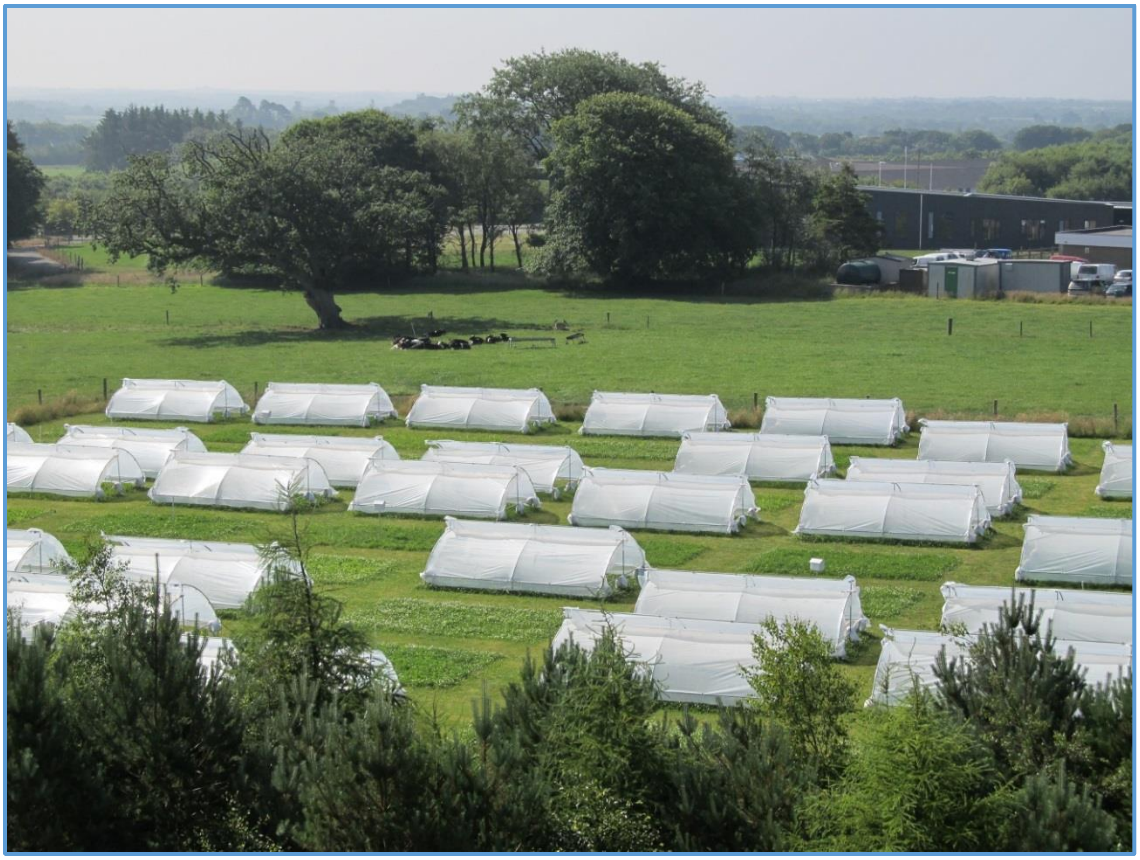

Fig. S4 Rain-out shelters in place on the field site, in July 2013 at Johnstown Castle, Co. Wexford, Ireland.

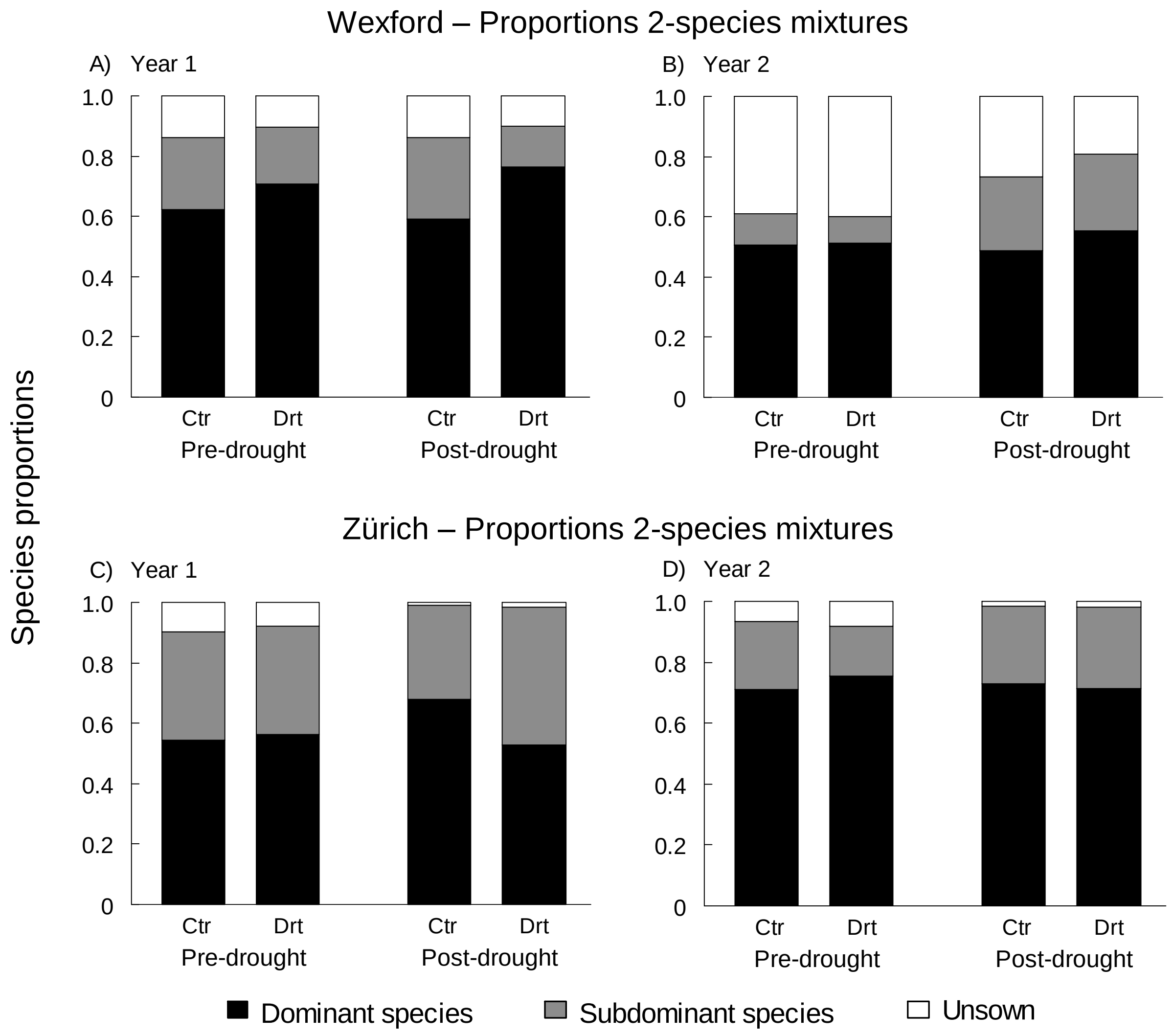

Fig. S5 Proportions of the dominant and subdominant species (plus the pooled unsown species) in harvested biomass yield, averaged across all two-species mixtures at Wexford (A, B) and Zürich (C, D) for the rainfed control (Ctr) and the drought treatment (Drt) during the pre-drought and post-drought period. The pre-drought and post-drought period comprised two and one harvest, respectively. Determination of the dominant species occurred at the pre-drought period and was held constant for post-drought.

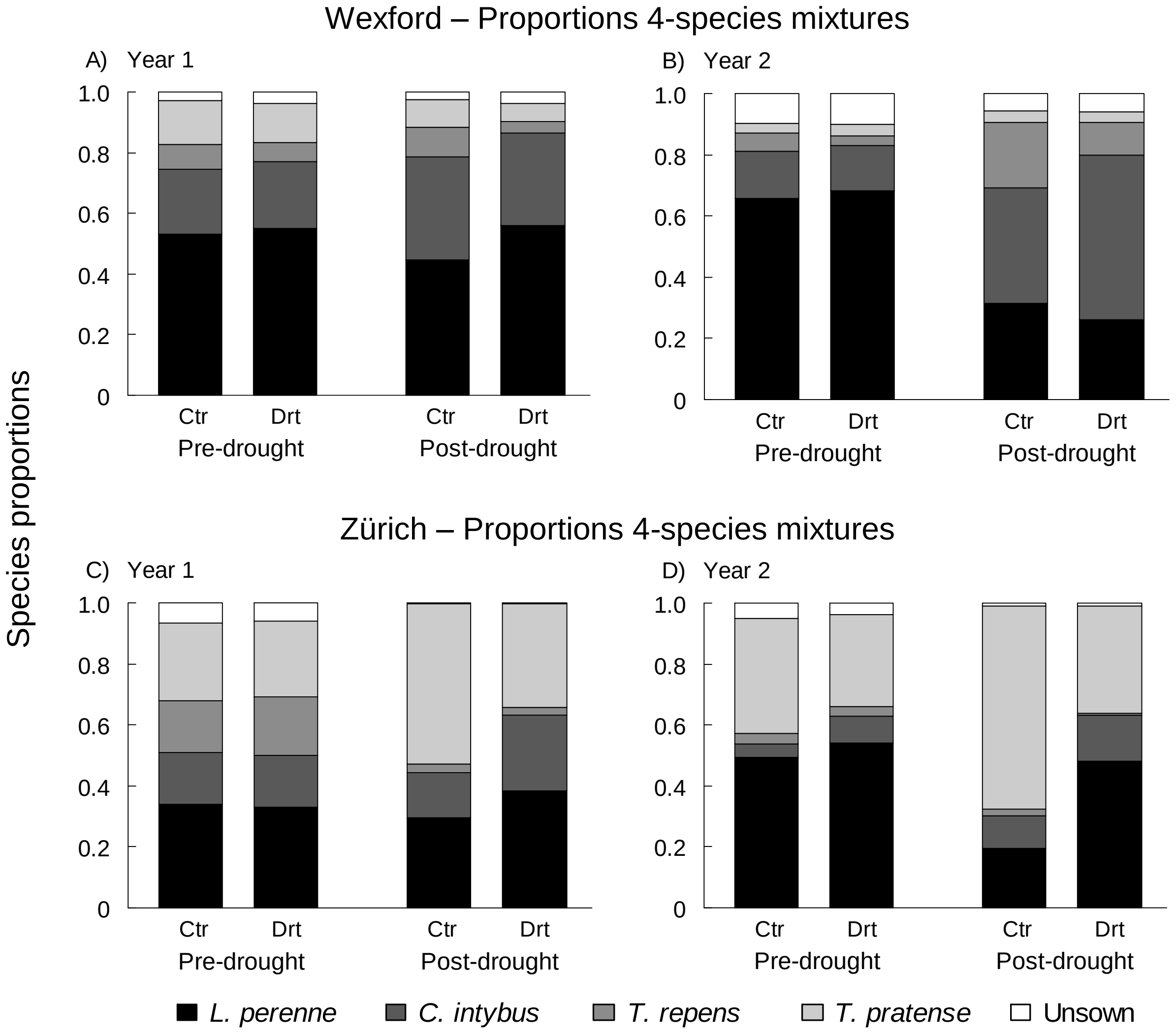

Figure S6. Proportions of sown species and the pooled unsown species in harvested biomass yield averaged across all four-species mixtures at Wexford (A, B) and Zürich (C, D) for the rainfed control (Ctr) and the drought treatment (Drt) during the pre-drought and the post-drought period. The pre-drought and post-drought period comprised two and one harvest, respectively.

Appendix S3 R code to equation 1

```
###############################
#Package to load

library(nlme)

###############################
#Reading in the data

d.data <- read.table("C:/Haughey et al 2018 DMY.csv", header=TRUE, sep= ";")

###############################
#Define factors

d.data$Harvest <- as.factor(d.data$Harvest)
d.data$Main_Plot <- as.factor(d.data$Main_Plot)
d.data$Split_Plot <- as.factor(d.data$Split_Plot)
d.data$Richness <- as.factor(d.data$Richness)

###############################
#Select one site

d.wexford <- subset(d.data, Site == "Wexford")
d.wexford <- droplevels(d.wexford)

###############################
```

#Full model, including all interactions, as described in eqn. 1 and the subsequent section. The
#variable "VarGroup" specifies the six groups *j* x *k* (three species richness levels *j* under control
#and drought conditions *k*), for which a (residual) variance was estimated

```
Model.A <- lme(DMY ~ Richness + Drt_Treat + Harvest + Richness:Drt_Treat +
               Richness:Harvest + Drt_Treat:Harvest + Richness:Drt_Treat:Harvest,
               random = ~ 1 | Main_Plot/Split_Plot,
               weights = varIdent(form = ~ 1 | VarGroup),
               contrasts = list(Harvest="contr.treatment"),
               method="REML", data=d.wexford)
```

#The regression summary inlcudes the estimated fixed effects (β parameters), the two random
#parameters λ_1 and λ_2, and the parameters δ_{jk}, out of which the six variances σ_{jk}^2 can be computed

```
summary(Model.A)

###############################
#Compute Table 2

anova(Model.A)

###############################
```

#The mean yields across all harvests can be computed as described in the main text with μ_{jk} = $^1/_m$ #$\sum_1^m y_{jkm}$ or can directly be received by specifying the contrast statement in a different form and #re-running the model with different order of levels for factors richness and drought

```
Model.B <- lme(DMY ~ Richness + Drt_Treat + Harvest + Richness:Drt_Treat +
               Richness:Harvest + Drt_Treat:Harvest + Richness:Drt_Treat:Harvest,
               random = ~ 1 | Main_Plot/Split_Plot,
               weights = varIdent(form = ~ 1 | VarGroup),
               contrasts = list(Harvest="contr.sum"), method="REML", data=d.wexford)
```
